# A Multifunctional 3D Co-Culture System for Studies of Mammary Tissue Morphogenesis and Stem Cell Biology

**DOI:** 10.1371/journal.pone.0025661

**Published:** 2011-09-30

**Authors:** Jonathan J. Campbell, Natalia Davidenko, Maria M. Caffarel, Ruth E. Cameron, Christine J. Watson

**Affiliations:** 1 Department of Pathology, University of Cambridge, Tennis Court Road, Cambridge, United Kingdom; 2 Department of Materials Science and Metallurgy, University of Cambridge, Cambridge, United Kingdom; University of California Merced, United States of America

## Abstract

Studies on the stem cell niche and the efficacy of cancer therapeutics require complex multicellular structures and interactions between different cell types and extracellular matrix (ECM) in three dimensional (3D) space. We have engineered a 3D *in vitro* model of mammary gland that encompasses a defined, porous collagen/hyaluronic acid (HA) scaffold forming a physiologically relevant foundation for epithelial and adipocyte co-culture. Polarized ductal and acinar structures form within this scaffold recapitulating normal tissue morphology in the absence of reconstituted basement membrane (rBM) hydrogel. Furthermore, organoid developmental outcome can be controlled by the ratio of collagen to HA, with a higher HA concentration favouring acinar morphological development. Importantly, this culture system recapitulates the stem cell niche as primary mammary stem cells form complex organoids, emphasising the utility of this approach for developmental and tumorigenic studies using genetically altered animals or human biopsy material, and for screening cancer therapeutics for personalised medicine.

## Introduction

**Figure 1:**
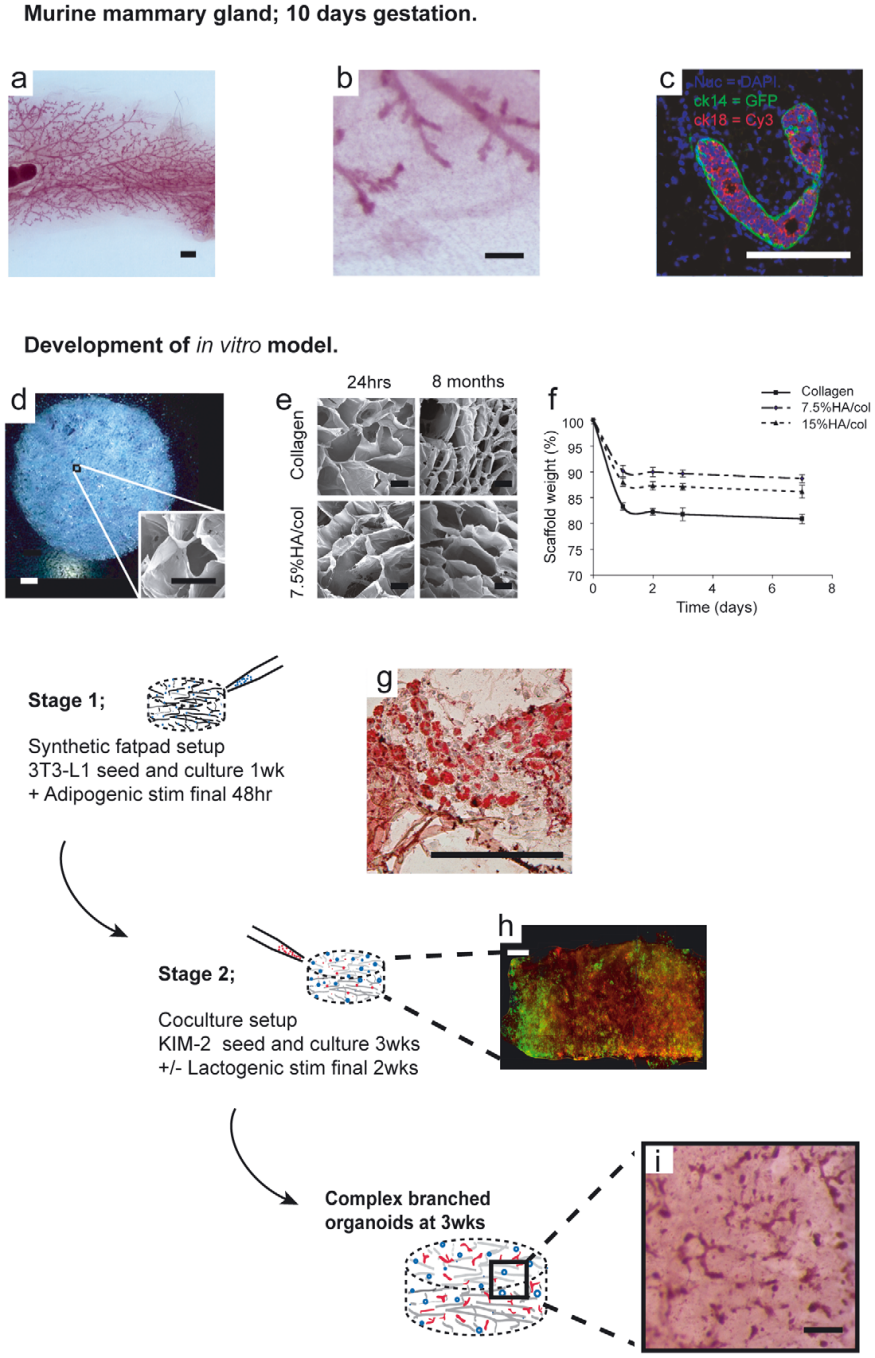
An in vitro strategy for recapitulating mammary gland architecture in 3D. (**a,b**) Whole-mount of murine mammary gland (10day gestation) showing branched epithelium invested within adipocyte-rich fat pad (bars 1mm and 200 µm respectively). Higher power immunohistochemical micrograph (**c**) of virginal gland showing distinctive architecture of luminal epithelial (cytokeratin 18+) and myoepithelial (cytokeratin 14+) bilayer (bar  = 200 µm). Phase contrast image (**d**) (bar  = 1mm) and S.E.M image (insert, bar  = 200 µm) of 7.5%HA/col scaffold showing interconnected pores that can support 3T3-L1 seeding and culture in 3D and differentiation to mature adipocytes. (**e**) S.E.M images of collagen and 7.5%HA/col scaffold following soaking in PBS for 24 hrs and 8 months showing collapse of porous structure in pure collagen scaffolds. (**f**) Stability of scaffolds by measurement of percentage weight retention during PBS incubation at 37°C. (**g**) 3T3-L1 seeded collagen scaffold following 8 days adipogenic culture. Haemotoylin/ Oil-red O staining (bar  = 200 µm). (**h**) Cross-section of thick (<2 mm) collagen scaffold following seeding with KIM-2 (GFP) for 48 hrs and 3T3-L1 (cell-tracker red) for 9 days (bar  = 500 µm). (**i**) Low-power observation of 3T3-L1/KIM-2 co-cultures (carmine stained) at 3 wks reveals branched epithelial structures with blunt ends (bar  = 200 µm).

The mammary gland is a useful model system for studying developmental processes such as branching morphogenesis and lineage commitment due to extensive post-natal development during puberty and successive cycles of remodeling during pregnancy, lactation and post-lactational regression (involution) [1]. Branching morphogenesis occurs initially by bifurcation of terminal end buds (TEB) during puberty to produce a ductal network (Fig. 1A, B) of bilayered epithelium consisting of luminal and myoepithelial layers, the latter in close contact with a laminin-rich basement membrane embedded within a collagen/adipocyte stroma (Fig. 1C). During pregnancy, tertiary branching and formation of lobuolaveolar milk producing structures (acini) takes place in response to estrogen, progesterone and prolactin (Prl). Such processes are largely dependent on the concerted movement of cells [2], often in response to reciprocal signaling between the epithelium and underlying mesenchyme. Such features should be recapitulated in the development of enhanced *in vitro* 3D models that support ductal-alveolar morphogenesis. However, current *in vitro* models such as Engelbrecht Holme-Swarm tumor-derived rBM or pure collagen gels do not utilize epithelial/stromal co-culture and display significant physiological and material limitations including tumor origin, batch variation and cell-mediated contraction altering porosity and elasticity of the gel structure [3] with implications for long-term studies. Indeed, model systems utilizing rBM could be considered more pertinent to end-stage developmental analyses highlighted by the tendency for mammary organoids to form cyst-like structures which fail to differentiate between ductal and alveolar mammary epithelium. ln this context it is noteworthy that most breast cancers arise in ducts [4].

Utilizing a controlled freeze drying and cross-linking procedure we have engineered a chemically defined porous scaffold matrix comprising two prevalent constituents of the mammary gland ECM, type I collagen and HA. Fibrous collagen type I is localized to mammary ducts [5], has a high tensile strength and numerous attachment sites for cells and biological mediators [6]. HA is a highly ionisable polysaccharide secreted to the pericellular space, where it contributes to tissue hydration and, through membrane bound receptors CD44 and RHAMM, influences cell motility [7], proliferation [8] and survival [9]. A morphogenic role for HA-CD44 signaling has been described in prostate [10], uretic bud [11] and mammary gland. HA concentration is both sensitive to exogenous estrogen and progesterone delivery [12] and is proportionally localized to the TEB [13]. We have thus sought to control epithelial organoid development *in vitro* of a novel bipotential progenitor mammary epithelial cell line KIM-2 [14] in co-culture with differentiated 3T3-L1 preadipocytes by varying the weight/% ratio of HA and collagen scaffold constituents. Unlike hydrogels, such a system supports seeding and differentiation of the stromal cell type by chemical mediators to form a synthetic fatpad prior to further 3D seeding with epithelial cells, recapitulating *in vitro* the migration of epithelium into stroma, a hallmark of mammary gland development.

## Results and Discussion

### An epithelial and stromal 3D co-culture strategy generates functional mammary tissue organoids

3T3-L1 preadipocytes have long been used as a model system to study adipogenic differentiation [15], are known to express high levels of two essential basement membrane proteins collagen type IV (colIV) and laminin [16] and have been shown to be important for mammary alveolar morphogenesis in 2D co-cultures [17]. KIM-2 cells are a conditionally immortal mouse mammary epithelial cell line that can give rise to both the luminal and myoepithelial lineages and differentiate to produce milk proteins by lactogenic hormone supplementation [14]. In 2D co-cultures of 3T3-L1 and KIM-2, *de novo* synthesis of basement membrane proteins is regulated by adipogenic differentiation and the localization of cell type (Fig. S1A-F). 3T3-L1 and KIM-2 mammary epithelial cells organized into monotypic islands (Fig. S1A) that further supported the differentiation of KIM-2 cells with lactogenic hormones (Fig. S1B). Copious laminin and colIV were localized to the stromal cell compartment (Fig. S1C,D). In transwell assays colIV protein levels were dependent upon adipogenic conversion of the 3T3-L1 compartment prior to seeding with KIM-2 cells (Fig. S1F). Furthermore, laminin protein levels were raised under adipogenic conditions when epithelial and stromal cell lines were overlaid for 7 days, thus demonstrating that direct contact between the epithelial and stromal cells is optimal.

Mindful of the well-recognized role of basement membrane in establishing appropriate epithelial polarity in mammary gland [18], we established a sequentially seeded stromal/epithelial co-culture model within a porous 3D scaffold matrix. Resultant collagen/HA scaffolds (Fig. 1D,E) exhibited homogenous intercommunicating pores mostly ranging in diameter between 100–300 µm. It was found that the addition of HA to collagen limited the collapse of the porous network with extended soaking (Fig. 1E), in addition to enhancing swelling and mass-loss properties of the scaffold compared to pure collagen scaffolds (Fig. 1F). To setup our *in vitro* system, stage 1 involves the generation of a 3D synthetic fatpad by immersing scaffolds in a diffuse suspension of 3T3-L1 cells. These cells are allowed to proliferate to confluence before inducing differentiation to mature adipocytes with standard adipogenic media supplementation (Fig. 1G). Stage 2 involves the introduction of KIM-2 cells throughout the scaffold, again via agitation within a cell suspension. Such a technique was found to be sufficient for diffuse seeding to full scaffold thickness (>1 mm, Fig. 1H, Fig. S3A) with both cell types located in direct contact. We had previously determined that short-term attachment of KIM-2 cells was enhanced by the presence of HA in thin film substrates of the same material as our porous scaffolds (Fig. S2). Cell viability studies quantifying the live-cell indicator calcein-AM versus the nuclear localization of propidium iodide (PI) in dead cells revealed that 3T3-L1 cells experienced a mean 14.3% reduction in viability over a 1 week period in pure collagen scaffolds against a mean 10.5% reduction within 7.5%HA/collagen scaffolds (Fig. S3B). This reduction in viability was matched across central and edge regions of each scaffold type, however cells cultured for longer periods (1 wk<) revealed that the prevalence of live cells was greater at the periphery of the scaffold (Fig. S3C), likely due to nutrient diffusion limitations to the scaffold centre. It is also notable that cell seeded scaffolds tended to float during the latter periods of culture (2 wks<), possibly due to increased adipose content.

**Figure 2:**
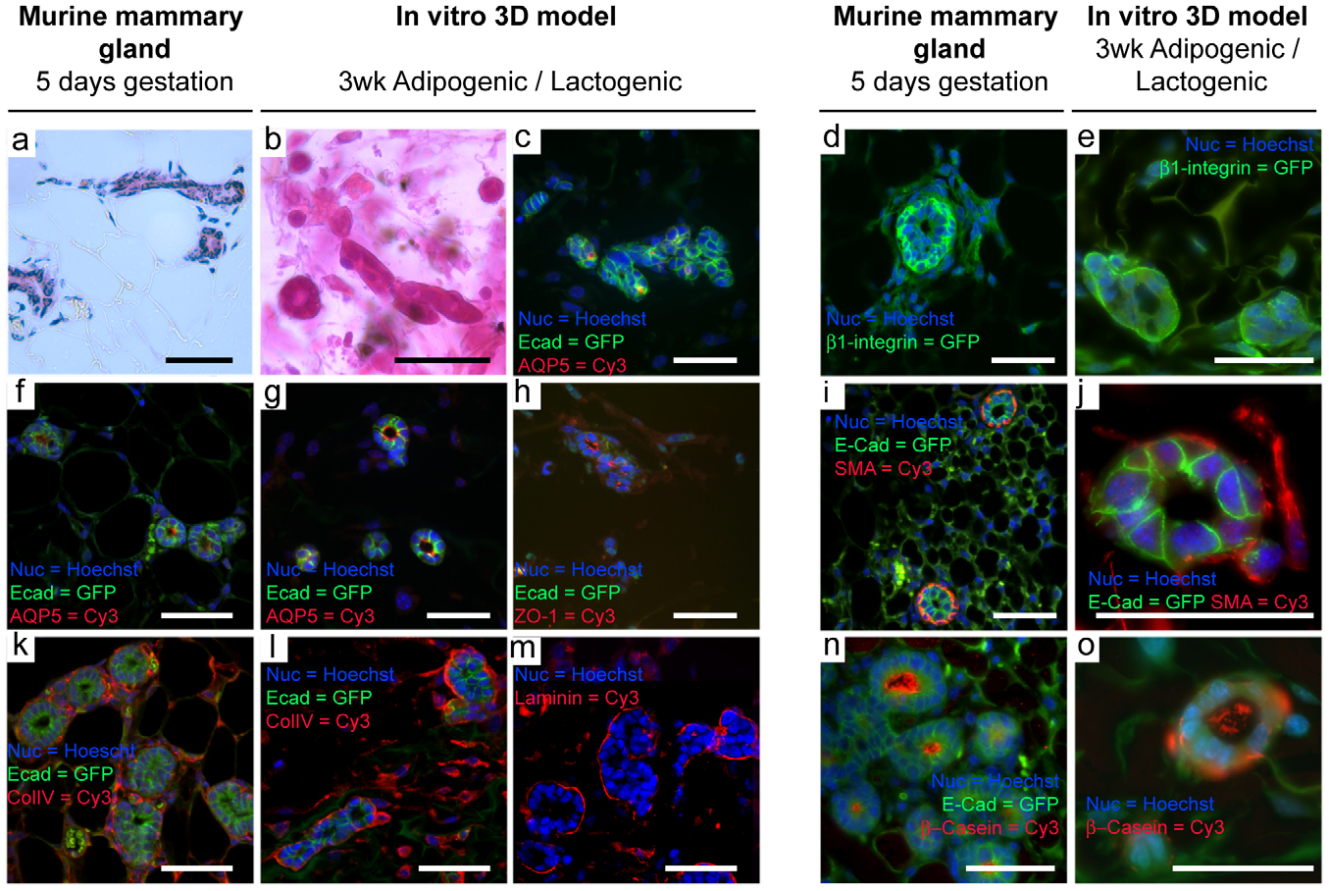
3T3-L1/KIM-2 co-culture in defined collagen/hyaluronic acid scaffolds generate bilayed, functional mammary epithelial organoids. H&E (a,b) and immunohistochemical (c-o) comparison of epithelial organoid formations *in vitro* with murine gland (scale bars  = 50 µm, except j,o  = 30 µm). Examples of branched ducts *in vivo* (a) and *in vitro* (b,c). Like the native gland at 5days gestation, epithelial organoids display polarity with AQP5 (g) and ZO-1 (h) towards a luminal cavity and integrin β-1 (e) and basement membrane constituents colIV (l) and laminin (m) at the basal surface. SMA(+) myoepithelial cells surround luminal epithelial cells (j), characteristic of the epithelial bilayer *in vivo* (i). With lactogenic stiumuli, organoid acinar-like structures express β-casein in a luminal cavity (o) as in the native gland (n).

Following 3 wks culture of KIM-2 cells in the presence (co-culture) or absence (mono-culture) of differentiated 3T3-L1 cells, and exposure to lactogenic hormone cocktail (+/-Prl) during the latter 2 wks (Lactogenic/Control), KIM-2 cells formed extensive branched organoids with blunt ends that exhibited ductal or acinar morphologies much like *in vivo* mammary gland (Fig. 1B,I, Fig. 2A-O). These organoids were predominantly located at a depth of 160 µm from the scaffold surface (Fig. S4). KIM-2 organoids displayed correct epithelial polarity with aquaporin 5 (AQP5) and ZO-1 tight junctions arranged towards a luminal cavity (Fig. 2 G,H respectively). Conversely integrin β-1 and colIV and laminin were localized to the basal surface (Fig. 2E, L, M respectively). Furthermore there was evidence that KIM-2 organized to an E-cadherin (E-cad) positive luminal epithelium surrounded basally by a smooth muscle actin (SMA) positive myoepithelium (Fig. 2J) in a similar manner to the native gland (Fig. 2I), and that β-casein is secreted and accumulates in a lumen in response to lactogenic hormones (Fig. 2O). These data demonstrate that functional polarized ductal and acinar structures can develop in the presence of a scaffold supported adipocyte ‘stroma’.

### Mammary organoid development can be directed through defined scaffold composition

**Figure 3:**
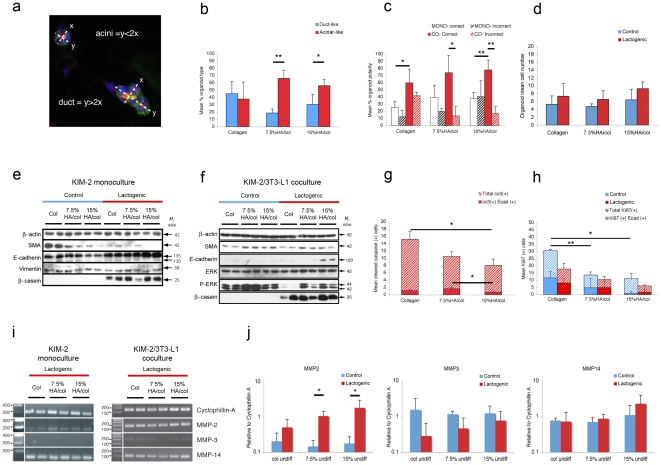
Mammary organoid fate is determined by scaffold composition. (**a**) Scoring of acinar or ductal AQP5(+)/E-cad(+) epithelial organoids determined by long axis (*y*)/short axis (*x*) ratios. (**b**) Inclusion of HA within scaffold composition significantly elevates frequency of acinar-like to duct-like organoids and (**c**) the frequency of correct organoid polarity is significantly elevated under co-culture (CO) as opposed to mono-culture (MONO) conditions and in the presence of HA (ANOVA, * and ** represent *p*<0.05 and *p*<0.01 respectively by post-hoc students T-Test, n>3). (**d**) Mean number of E-cad(+) KIM-2 per organoid, measured at constant focal plane of immunohistochemically prepared sections. Western analysis of KIM-2 mono-culture (**e**) and KIM-2/3T3-L1 co-cultures (**f**) under KIM-2 maintenance media (control) or exposure for latter 2 wks to lactogenic media (lactogenic). (**g**) Inclusion of HA at 15% reduces cleaved caspase 3(+) cells and limits cell proliferation (**h**) at 7.5% and 15% compared to collagen control under lactogenic conditions by measurement of total Ki67(+) cells (* and ** represent *p*<0.05 and *p*<0.01 respectively by students T-Test). (**i**) PCR analysis of *MMP2, MMP3* and *MMP14* gene expression within 3D scaffolds of 3 wk KIM-2 mono-culture lactogenic specimens and 3T3-L1/KIM-2 co-culture lactogenic specimens. (**j**) Quantitative real-time PCR analysis of *MMP2, MMP3* and *MMP14* gene expression normalized to *Cyclophillin A*. Bars represent mean of at least 3 separate experiments performed in triplicate. Error bars represent SD. * represents statistical significance by students T-test (*p*<0.05)

To assess whether the scaffold can be modified to manipulate epithelial developmental outcome, dual positive AQP5/E-cad KIM-2 organoids were scored morphologically as ducts or acini by analyzing long and short axis ratios (Fig. 3A). Strikingly, HA-incorporated scaffolds yielded organoids with proportionally more acinar-like characteristics with 7.5%HA being optimal (Fig. 3B). In addition, E-cad(+) organoids were scored for physiological polarity based on ‘correct’ centrally localized AQP5 versus an ‘incorrect’ peripheral localization. Under lactogenic stimuli, the presence of 3T3-L1 cells enhanced the frequency of physiological KIM-2 organoid development, where the further addition of scaffold HA ensured correctly polarized outcome (Fig. 3C). Further analysis through immunoblotting, revealed that HA increased E-cad expression in lactogenic co-culture (Fig. 3F) and in its precursor form in mono-culture conditions (Fig. 3E). A concomitant decrease in SMA expression in mono-culture conditions with no accompanying modulation of vimentin expression possibly indicated a mesenchymal-epithelial transition (MET) (Fig. 3E). This diminution of basal cells is supported by flow cytometric analysis of CD24/CD49f which revealed a small but significant reduction in the percentage of myoepithelial KIM-2 cells grown on 15% HA/col films compared to pure collagen and 7.5%HA/col films (Fig. S5).

Strikingly, phosphorylated ERK was elevated within 3D 7.5% HA/col scaffold co-cultures and maintained within HA scaffolds upon lactogenic differentiation (Fig. 3F). Sustained activation of ERK signaling is required for mammary epithelial branching morphogenesis [19], whilst inhibiting its action limits branching but allows tubule elongation in developing kidney [20]. In addition ERK activation has been shown to limit mammary luminal cell death during mammosphere formation [21]. An analysis of cleaved caspase 3 (cc3) activity revealed 15% HA/col cultures exhibited a lower apoptotic index compared to pure collagen scaffolds. Limiting the analysis to dual positive AQP5/E-cad cells demonstrated a similar reduction in 15%HA/col compared to 7.5%HA/col (Fig. 3G). It is worth noting, however, that there was no evidence of apoptotic cells within KIM-2 luminal cavities, possibly indicating a mechanism of localized polarity and cell rearrangement forming hollow organoids in our system. Furthermore, whilst the presence of HA resulted in a reduction in total cell mitotic index in the absence of lactogenic stimuli (Fig. 3H), there was no observable reduction in organoid size (Fig. 3D). This may be a consequence of enhanced cell migration, observed in wound-closure assays on HA/col films (Fig. S6). Matrix-metalloproteinases (MMPs) -2, -3 and -14 play an important role in branching morphogenesis, demonstrating compartmentalized expression between the epithelium and surrounding stroma [22] and recently have been implicated in mammary epithelial motility and organization [23]. Interestingly lactogenic co-culture conditions enhanced MMP-2 gene expression over mono-culture (Fig. 3I) and was further enhanced by HA (Fig. 3J).

HA has been implicated in numerous biological interactions and its influence on mammary organoid development in the current study is likely a result of multifarious chemical and physical interactions. As well as interacting with a diverse number of hyaloronan-binding proteins [24], its biological effect is known to be dependent on chain length [25], with shorter oligosaccharides shown to regulate angiogenesis [26], EMT [27] and inflammation [28]. HA is synthesized at the cellular surface by membrane associated HA synthases (HAS1-3) where it can form a substantial pericellular coat with gel-like properties [29] and is known to govern early (<minute) attachment to ECM prior to integrin tethering and adhesion maturation [30]. This interaction is known to be attenuated by treatment with hyaluronidase or the presentation of an HA coated surface [31]. Importantly, there was no decreased rate of cell attachment in the present study in the presence of HA, although this was assessed over a timescale suitable to establish integrin-mediated focal adhesions. However, considering the focal nature of integrin attachment [32] it is possible that the increased frequency of acinar-like structures in HA scaffolds may be attributed to localized electrostatic repulsion of HA coated cells and scaffold surfaces favouring rounded organoid formation, interspersed with integrin mediated adherence to regions of collagen type I.

### Primary organoid development and stem cell enriched populations are supported using a synthetic fatpad *in vitro*

**Figure 4:**
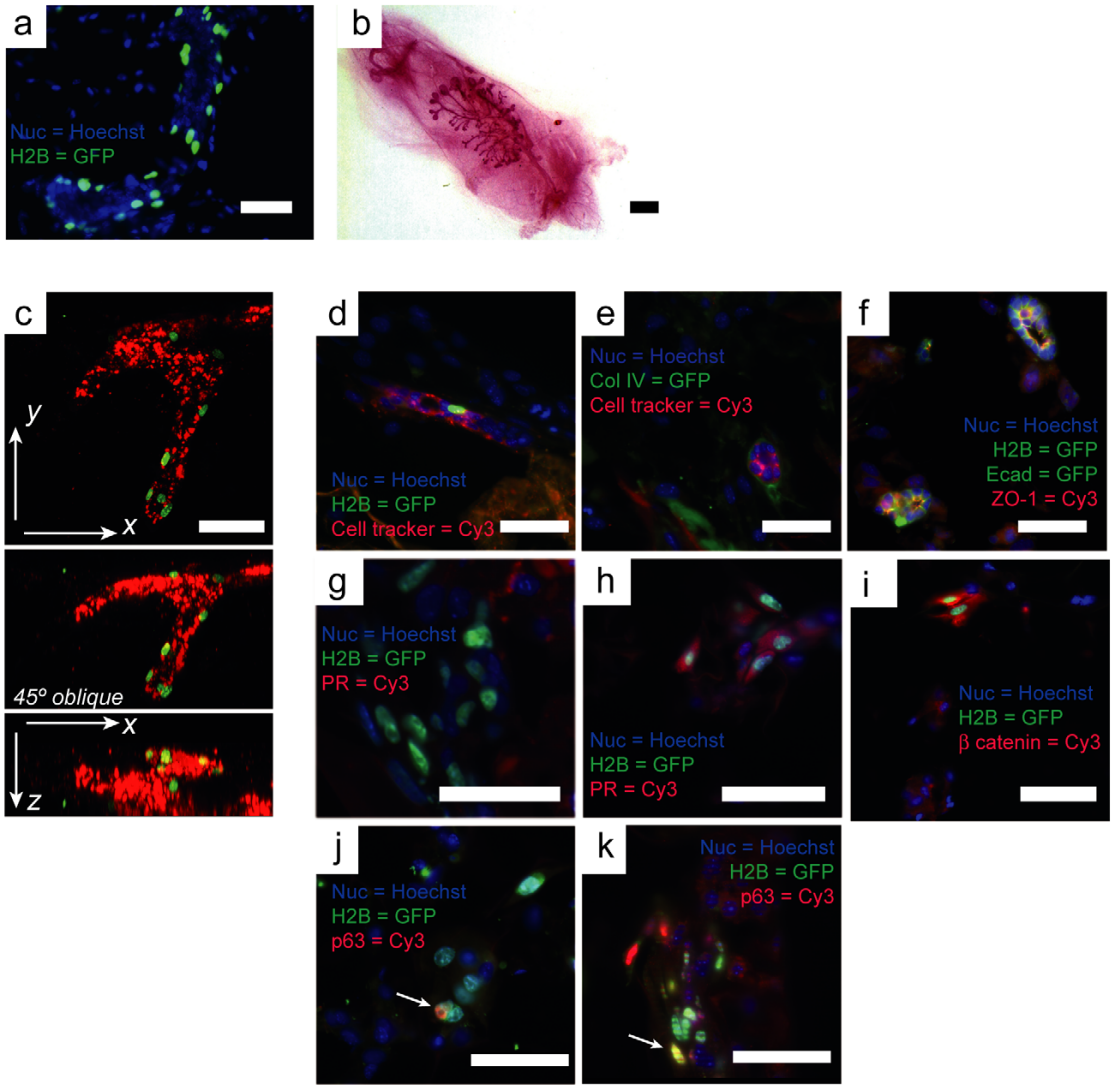
3T3-L1 seeded collagen/HA scaffolds support primary epithelial cell organoid development and provide a stem cell niche. (**a**) 5 day gestation section of murine mammary gland expressing GFP-tagged histone H2B under the control of the Keratin 14 promoter. Cells of the myoepithelial/basal lineage are green while luminal cells do not express H2B-GFP. (**b**) Stem cell enriched (GFP+, CD24^med^ CD49f^high^) fractions of these cells are capable of mammary gland regrowth following transplantation to a cleared fatpad. (**c**) Live cell confocal image representation (orthogonal view and oblique projection) of primary cell culture following 10days co-culture with 3T3-L1 showing basal localization of GFP+ cells around a branched organoid. Primary epithelial organoids co-cultured with 3T3-L1 in pure collagen (d,e) and 7.5%HA/col (f) scaffolds. Organoids form ductal and acinar-like structures enveloped in basement membrane (**e**) and exhibit CK14 basal cells arrayed around a clear lumen with correctly polarized E-cad (+) luminal epithelial cells expressing ZO-1 at the apical cell surface (**f**). (**g,h**) Sorted GFP+ label retaining cells form colonies that vary in their expression of cytoplasmic progesterone receptor (PR), and are beta catenin positive (**i**). (**j,k**) Colonies display positive and negative staining for p63 suggesting self-renewal or asymmetric cell division (indicated by arrows) (Bars  = 100 µm).

The ability to grow primary mammary cells in our scaffolds would have substantial benefit. Firstly, such cells enable more relevant physiological studies such as lineage commitment and plasticity. In addition, cells from genetically altered mice could be used to allow investigation of gene function in 3D. The use of our adipocyte seeded scaffolds could also replace the mammary fat pad for stem cell transplantation studies. We therefore replaced KIM-2 cells with primary cells isolated from a transgenic mouse expressing a GFP-tagged histone H2B [33] expressed under the control of the Keratin 14 (K14) promoter, which is expressed in basal/myoepithelial cells but not in luminal (K18 expressing) cells *in vivo* (Fig. 4A). These animals had been fed with doxycyclin for 1 week prior to harvesting of mammary tissue in order to restrict GFP expression to the slower cycling stem cell compartment. Further labeling of the primary cell isolate with a fluorescent tracer dye prior to scaffold seeding enabled the real-time visualization of large branched organoids following 10 days co-culture with 3T3-L1 (Fig. 4C). Such structures exhibited basally localized GFP, in a similar manner to that of the native gland, and clustering of GFP+ cells at distal extremities. Other researchers have reported a similar localization of basal cells toward the distal tips of epithelial outgrowths *in vivo* [34]. Immunohistochemistry revealed organoids with cleared lumens, surrounded by basement membrane and appropriate polarity confirmed by the expression of ZO-1 toward the apical surface (Fig. 4D-F).

We next sought to determine whether our 3D scaffolds would support mammary epithelial niche formation and substitute for fat-pad transplant experiments by sorting cells for GFP expression and co-culturing pure GFP+ populations with differentiated 3T3-L1. Following 2 wks of culture, GFP+ cells aggregated, displaying varying levels of GFP, beta-catenin and cytoplasmic localization of progesterone receptor (Fig. 4G,H,I respectively). Interestingly, GFP+ cells displayed a heterotypic p63 profile (Fig. 4J,K), probably indicating asymmetric cell division. It is known that p63 functions as a molecular switch during the stratification of epithelial tissues [35], and this may demonstrate the initiation of myoepithelial differentiation from a progenitor pool.

### Conclusions

We have developed an *in vitro* model of mammary gland that allows for the maintenance and differentiation of both epithelial and stromal derived cell lines within a defined 3D scaffold resulting in tissue formation comparable to the native gland. The major advantage of such a system is that it does not require the presence of tumor-derived rBM and retains a stable 3D environment for extended periods in culture. In addition the utility of our scaffolds has been confirmed through data demonstrating that its composition influences mammary organoid developmental outcome in addition to supporting primary stem and progenitor cell organoid development with both ductal and acinar morphology. This study presents a framework for the further development of defined 3D model systems compatible with diverse modification through spatial, biochemical and mechanical control, that may support and direct tissue development from human primary cells. Ultimately this will lead to a better understanding of developmental and disease processes, and allow for personalized medicine initiatives through tailored assays of breast biopsy material and high-throughput therapeutic drug screening.

## Materials and Methods

All animal work was carried out in accordance with the rules and guidance of the University of Cambridge local ethical committee and a project license awarded by the UK Home Office. All materials were obtained from Sigma-Aldrich, Poole, UK, unless otherwise stated.

### Collagen/hyaluronic acid scaffold and film fabrication

Highly porous scaffolds were produced from a collagen-HA suspension using a freeze-drying technique. Firstly, a suspension of 1%weight (wt) of different component compositions were prepared from bovine type I collagen from Achilles tendon and bovine HA from vitreous humor (H7630, Sigma) in a 0.05 M pH 2.0 acetic acid solution. Each suspension was blended at 20,000 rpm using an overhead homogenizer for 30 min at 40°C. After mixing, the suspension was debubbled by centrifugation at 2500 rpm for 5 min. Two different HA weight percentages (7.5 and 15 wt-%) and pure collagen suspensions were produced which were then frozen in 316L stainless steel plates at a controlled rate to −30°C and subsequently sublimed at 0°C for 24 h under a vacuum of less than 100 mTorr, using a computer-controlled freeze-dryer. Lyophilized collagen and collagen-HA scaffolds were crosslinked in 95% ethanol solution containing 33 mM of 1-ethyl-3-(3-dimethylaminopropyl)-carbodiimide hydrochloride (EDC) and 6 mM of N-hydroxysuccinimide (NHS) for 4 h at 25°C. After the crosslinking, the scaffolds were washed thoroughly in distilled water (5 min×5) and were subsequently re-frozen and re-lyophilized using the previous freeze-drying cycle. Disc samples of 10 mm diameter were then cut from lyophilized sheet using a punch. Films were prepared from different collagen-HA 0.5%-wt suspensions using the same procedure. Suspensions of collagen, 7.5%HA/col and 15%HA/col were subsequently spread over 60 mm Petri dishes at a quantity suitable to obtain films of 5–7 µm thickness (assuming a density of solid collagen at 1.3 g cm^−3^ and of HA 1.0 g cm^−3^). Dried films were cross-linked with a water-soluble carbodiimide EDC in the presence of NHS following the same procedure as for scaffold production, and then re-dried in air. Before cell culture, scaffolds and films were sterilized by exposure to UV light at 500 mJ/cm^2^ and rinsed trice in distilled water and phosphate buffer saline (PBS) before incubation in PBS for 24 h.

### Direct and transwell co-culture of 3T3-L1 and KIM-2

3T3-L1 cells (ATCC, Manassas, VA, USA) were plated at 3×10^4^/cm^2^ in 12 well plates and maintained for 5days in DMEM + 10% new born calf serum (NBCS) (both Gibco, Invitrogen Paisley, UK). Terminal differentiation was induced by treatment for 48 h with DMEM, 10% fetal calf serum (FCS, Gibco), l µM dexamethasone (DEX), 0.5 mM 3-iso-butyl-1-methylxanthine (IBMX) and 0.01 mg/ml^−1^ insulin. Following this procedure, 3×10^5^ KIM-2 cells [14] were added to each 3T3-L1 seeded well (Direct culture) or to transwell inserts positioned above the 3T3-L1 confluent cell sheet and each well was maintained in KIM-2 maintenance media consisting of DMEM+F12 1∶1 + 10% FCS, 0.8 mM insulin, 0.8 mM EGF and 17 mM linoleic acid (LA). Co-cultures were maintained for a period of 1 wk before analysis. For confirmation of lactogenic differentiation by KIM-2 in the presence of 3T3-L1, 1 well was rinsed in PBS and bathed in lactogenic media, DMEM+F12 1∶1 plus 10% FCS, 0.8 mM insulin, 0.2 mM prolactin, 1 mM DEX and 17 mM LA for a further week.

### Mono-culture and Co-culture preparation of 3D cell-seeded scaffolds

The 3T3-L1 cell line (ATCC) was maintained in DMEM plus 10% NBCS, and passaged at <80% confluence. KIM-2 cells were passaged in KIM-2 maintenance media as described above. To seed 3D co-cultures, a single cell suspension of 3T3-L1 cells was adjusted to 10^6^ cells/mL in a 50 ml falcon tube. Five scaffolds were added to a 10 mL cell suspension, each scaffold being rolled against the tube sidewall using a sterile pipette to eliminate air bubbles. Cell/scaffold suspensions were removed to a rotary plate set to 0.5 Hz for 4 h at 37° to allow further penetration of cells to the scaffold centre. Cell-seeded scaffolds were then removed to a 12 well plate and incubated in 1.5 mLs DMEM plus 10% NBCS for 5 days to ensure cell confluence, confirmed by live cell fluorescence visualization with 5 µM calcein-AM (Invitrogen). Terminal differentiation was induced for 48 h as previously described prior to secondary seeding with epithelial cells. Secondary seeding of KIM-2 cells was accomplished using the same techniques but without compression of the scaffolds. Co-cultures were maintained in KIM-2 maintenance media for 1 wk before rinsing with PBS then treatment with lactogenic media, DMEM+F12 1∶1 plus 10% FCS, 0.8 mM insulin, 0.2 mM prolactin, 1 mM DEX and 17 mM LA for 2 wks. KIM-2 mono-cultures were established using the same method and grown for 3 wks total, with or without 2 wks lactogenic culture. Following incubation periods cell seeded scaffolds were snap frozen in liquid N_2_ or fixed with 4% paraformaldehyde for cryosectioning and paraffin embedding. Whole cell–seeded scaffolds were stained with carmine following previously published methods [36]. Cut sections were stained with H&E and Oil-Red O as described previously [37].

### 3D Co-culture of 3T3-L1 and primary murine mammary epithelial cells

Tissue from the mammary glands of H2B-GFP expressing transgenic mice [33] was digested overnight at 37°C in DMEM + F12 containing 1 mg/mL collagenase (10103578001, Roche, Switz) and 1000 U/mL hyaluronidase and further dissociated with 5 mg/ mL dispase, 0.1 mg/mL DNase, and trypsin-EDTA. Red blood cells were removed with ammonium chloride. These cells were then adjusted in suspension to 10^5^ cells/mL and seeded within 1 wk adipogenic 3T3-L1 seeded scaffolds. Primary epithelial seeded co-cultures were distributed within 12 well plates and maintained for a further week in 1.5 mL KIM-2 maintenance media, with or without progesterone (10 nM). Specimens were then processed as above. For the selection of H2B GFP+ cells, freshly digested primary tissue isolates were sorted on the GFP channel of a MoFlo cell sorter (Beckman Coulter, USA). Propidium iodide (PI, 2.5 µg/ml) was added one minute before sample analysis.

### KIM-2 attachment and migration on 2D films

A KIM-2 cell suspension of 10^6^ cells/mL was incubated in the presence or absence of anti-CD44 (1/500, KM114, BD Biosciences, Oxon UK.) for 30 m prior to seeding on collagen, 7.5%/HAcol and 15%HAcol films at 10^5^ cells/cm^2^ and monitored by phase-contrast microscopy for 24 hrs. Cells were judged to be attached by visual observation of cell flattening and lamellopodia formation. At 48 hrs a confluent cell surface was scratched using a sterile pipette and migration monitored at 10minute intervals using time-lapse phase-contrast microscopy at x100 (Zeiss Axiovert S100, Carl Zeiss, Herts, UK). Individual cell movement was monitored by tracking cell nuclei using ImageJ software (Rasband, W.S., ImageJ, U. S. National Institutes of Health, Bethesda, Maryland, USA, http://rsb.info.nih.gov/ij/, 1997–2009).

### Basal and luminal epithelial FACS analysis on 2D films

Epithelial cells were trypsinized and labeled with the following antibodies: PE anti-CD24 (1/500 , 12-0242, eBioscience, Hatfield, UK) and Alexa Fluor 647 anti-CD49f (1/200, 313610, BioLegend, Cambridge, UK). Propidium iodide (PI, 2.5 µg/ml) was added one minute before sample analysis and cells were filtered at 30 µm immediately prior to FACS. Cells were analyzed using a CyAnTM ADP flow cytometer (Dako, Glostrup, Denmark). Ten thousand cells per analysis were recorded.

### Immunodetection

Immunohistochemistry was carried out on Paraffin-embedded and cryopreserved sections. Paraffin-embedded samples were de-pariffinized and rehydrated before antigen retrieval by boiling in 10 mM Tri-sodium citrate buffer pH 6.0 for 11 m. Sections were blocked for 1 hr in 10% normal goat serum (NGS) in Tris buffer saline + 0.1% Tween20 (TBST) prior to incubation at 4°C overnight in primary antibodies or isotype controls. Antibodies were prepared at the following dilutions in 10% NGS + TBST; E-cad (1/500, 610182, BD Biosciences), β-casein (a gift from Bert Binas, 1/500), ZO-1 (1/250, MAB 1570, Millipore Temicula CA, USA), β-1 integrin (1/100, MAB 1997, Millipore), AQP5 (1/100, AB3559, Millipore,), SMA (1/200, ab5694, Abcam, Cambs UK), Ki67 (1/500, NCL-Ki67p, Leica Biosystems, Newcastle UK), Laminin (1/100, ab11575, Abcam), Collagen IV (1/100, ColIV, ab6576, Abcam), Cleaved-caspase 3 (1/100, CC3, 9661S, Cell Signalling, Beverly, MA USA). Signal was detected using Cy3- and Alexa-488 (Invitrogen) conjugated secondary antibodies (1/400) and nuclei stained with bisbenzimide-Hoechst 33342 before analysis by fluorescence microscopy and data capture (Zeiss Axiophot). Immunocytochemistry was carried out on cell monolayers fixed for 4 hrs in paraformaldehyde and permeabilized with 0.25%Triton X-100 before blocking in blocking buffer and antigen detection as described above. Preparation for immunoblotting and signal detection was carried out as previously described [36] with lanes adjusted for equal protein loading by densitometric lane analysis of β-actin (Abcam, Cambs UK) using ImageJ software. Antibodies against β-casein (1/10000), E-cad, SMA (ab5694, Abcam). Vimentin (V4630), P-ERK (9106S, Cell Signalling), ERK (610124, BD Biosciences) (all 1/1000) were used to probe against specific antigens.

### Live cell imaging

Whole primary epithelial and GFP+ sorted cell isolates were loaded with 10 µM cell tracker dye (Celltracker red CMTPX, Invitrogen) for 30 minutes prior to seeding in adipogenic scaffolds as outlined above. Following 10 days of culture, scaffolds were removed to the environment chamber of a confocal microscope (Leica TCS SP II, Leica, Germany) and GFP and cell tracker visualised by laser excitation at 488 nm and 543 nm respectively. Images were analyzed by propriety software (Leica). For an assessment of sequential 3D seeding, 3T3-L1 cells loaded with cell tracker dye were cultured in scaffolds for 48 hrs prior to seeding with KIM-2 cells transfected with an MSCV-IRES-GFP empty vector (kindly provided by Prof. Göttgens, Department of Haematology, Cambridge, UK).

### Viability testing

3T3-L1 seeded scaffolds were halved in the vertical plane and bathed for 1 hr in complete media with 5 µM Calcein AM (Invitrogen). PI was added for the final 5 minutes of incubation (2.5 µg/ml) to label dead cells. Scaffolds were placed cut-side down on a glass coverslip and viewed under a fluorescent microscope using a 20x objective and digital image capture.. Live and dead cells were recorded using ImageJ software under a 300% zoom factor. Edge measurements corresponded to within 1 imaging frame of the scaffold edge (maximum depth of 185 µm).

### Image analysis and statistics

Quantification of Ki67 and CC3 signal and pixel measurement of long and short axis ratio in E-cad+/AQP5+ organoids was performed using ImageJ software point selection and line selection tools respectively. Confocal image analysis was performed using propriety software (Leica). All statistical analyses by *a priori* ANOVA and student T Test were performed using Excel data analysis toolpak (Microsoft, WA, USA).

### RNA extraction and PCR of gene expression analysis

Total RNA from snap-frozen cell seeded scaffolds was extracted using TRIzol reagent (Invitrogen), precipitated in isopropanol and purified using an RNeasy mini kit (Qiagen, Crawley UK). RNA was quantified using a Nanodrop ND-1000 (Nanodrop products, Wilminton DE, USA) spectrophotometer and cDNA was reverse transcribed from 1 µg of RNA using random hexanucleotide primers (Promega, Madison, WI, USA) and superscript III reverse transcriptase (Invitrogen). Gene expression of *MMP-2, MMP-3 and MMP-14* and *cyclophillin A* was detected by semi-quantitative methods using Taq DNA polymerase (Qiagen) and real-time quantitative methods by SYBR-green dye chemistry by following the manufacturers protocol. Real-time PCR reactions were run on an iCycler (Biorad) in triplicate and quantitative analysis performed using iCycler iQ Real-Time Detection Software (Biorad) normalized to *Cyclophillin A*, FW; CCTTGGGCCGCGTCTCCTT, RV; CACCCTGGCACATGAATCCTG. The following primer sequences were obtained using the PrimerBank [38] website (http://pga.mgh.harvard.edu/primerbank/): *MMP-2*, FW; CAAGTTCCCCGGCGATGTC, RV; TTCTGGTCAAGGTCACCTGTC, *MMP-3*, FW; ACATGGAGACTTTGTCCCTTTTG, RV; TTGGCTGAGTGGTAGAGTCCC and *MMP-14*, FW; CAGTATGGCTACCTACCTCCAG, RV; GCCTTGCCTGTCACTTGTAAA. All primers were obtained from Sigma.

## Supporting Information

Figure S1 **Basement membrane synthesis supported by adipogenic differentiation of 3T3-L1 and localization with KIM-2 cells.** In a 2D co-cultures, stromal 3T3-L1 (*s*) and epithelial KIM2 cells (*e*) form distinct regions (**a**). With the addition of lactogenic hormone prolactin (prl) KIM-2 cells formed characteristic acinar-like regions (*ac*) (**b**). Two essential basement membrane proteins laminin (**c**, pan-laminin red channel, cytokeratin 18, green channel) and collagen IV (colIV) (green channel, **d**) are localized to the stromal compartment (s). A transwell culture model (**e**) reveals that colIV protein expression is dependent of adipogenic stimuli and laminin is upregulated through direct contact of KIM2 and 3T3-L1 cells (**f**). F–J bar  = 50 µm.
(TIF)

Figure S2 **Attachment KIM-2 cells to fabricated films.** Percentage KIM-2 attachment to 2D collagen and collagen/HA films with and without the addition of anti-CD44 blocking antibody.
(TIF)

Figure S3 **Seeding and viability of cells in 3D scaffolds.** (**a**) Full depth seeding of 3T3-L1 preadipocyte cell suspensions in collagen scaffold (bar  = 100 µm). (**b**) Viability assessment within cross section of 3T3-L1 seeded collagen scaffold following 12 days of culture (green  =  calcein AM, red  =  PI). (**c)** Mean 3T3-L1 viability at indicated times within collagen and 7.5%HA/col scaffolds. Live and dead cells were counted within edge (to a depth <185 µm) and centre regions using ImageJ software. Error bars represent standard deviation.
(TIF)

Figure S4 **3D epithelial organoid formation towards the scaffold surface.** Confocal live-cell images at indicated depths (z dimension) of KIM-2 mono-cultures in 7.5%HA/col scaffold following 1 wk of culture in maintenance media (cells labelled with cell tracker red with look-up table imaging, Leica confocal software)
(TIF)

Figure S5 **Analysis of luminal and basal mammary epithelial cells cultured on fabricated films.** Representative FACS dot plots of KIM-2 cells cultured for one week on plastic or on 2D films of collagen, 7.5%HA/col or 15%HA/col and stained with CD24 and CD49f antibodies to separate luminal (CD24^high^ CD49f^med^) and basal (CD24^med^ CD49f^high^) populations. The mean % +/- SD of basal cells within the total cell population in each condition is shown.
(TIF)

Figure S6 **Cell migration analysis on fabricated films by wound closure assay.** Representative images of scratch assays at indicated times on collagen and 7.5%HA/col films. Yellow line indicates migratory front of cells. Plotted velocities of KIM-2 cells µm/min^−1^.
(TIF)
