# Supplementary figures and images for: A Multifunctional 3D Co-Culture System for Studies of Mammary Tissue Morphogenesis and Stem Cell Biology
Source: PLoS One. 2011 Sep 30;6(9):e25661. doi: 10.1371/journal.pone.0025661 (PMC3184152; doi:10.1371/journal.pone.0025661)

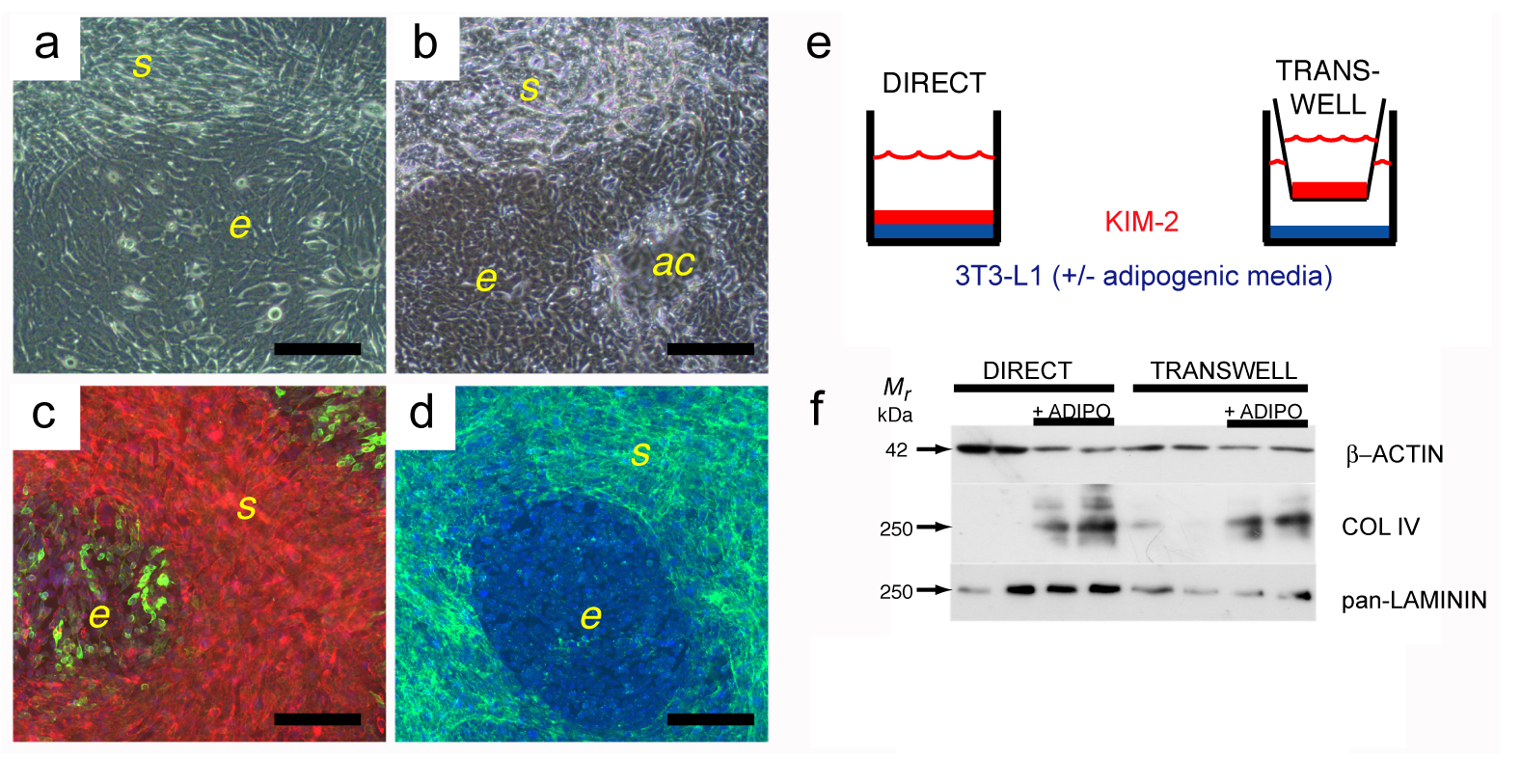

Supplement: Figure S1 — Basement membrane synthesis supported by adipogenic differentiation of 3T3-L1 and localization with KIM-2 cells. In a 2D co-cultures, stromal 3T3-L1 (s) and epithelial KIM2 cells (e) form distinct regions (a). With the addition of lactogenic hormone prolactin (prl) KIM-2 cells formed characteristic acinar-like regions (ac) (b). Two essential basement membrane proteins laminin (c, pan-laminin red channel, cytokeratin 18, green channel) and collagen IV (colIV) (green channel, d) are localized to the stromal compartment (s). A transwell culture model (e) reveals that colIV protein expression is dependent of adipogenic stimuli and laminin is upregulated through direct contact of KIM2 and 3T3-L1 cells (f). F–J bar = 50 µm. (TIF) [file pone.0025661.s001.tif]

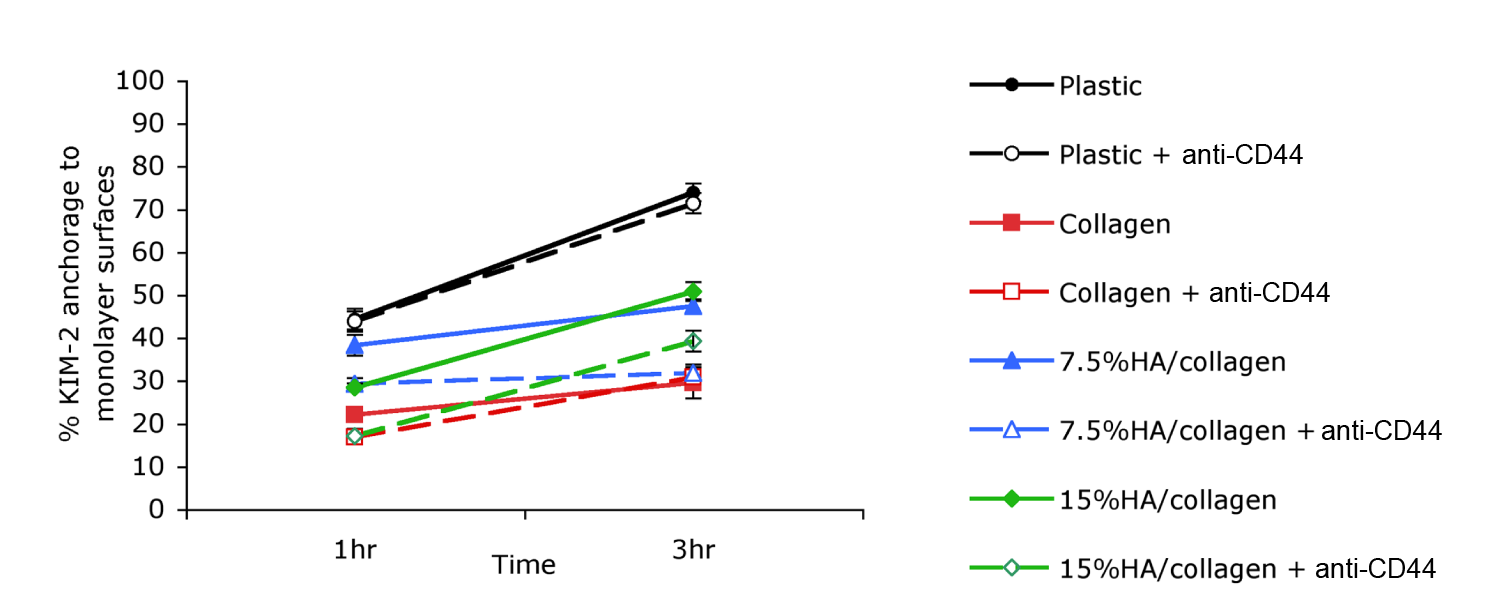

Supplement: Figure S2 — Attachment KIM-2 cells to fabricated films. Percentage KIM-2 attachment to 2D collagen and collagen/HA films with and without the addition of anti-CD44 blocking antibody. (TIF) [file pone.0025661.s002.tif]

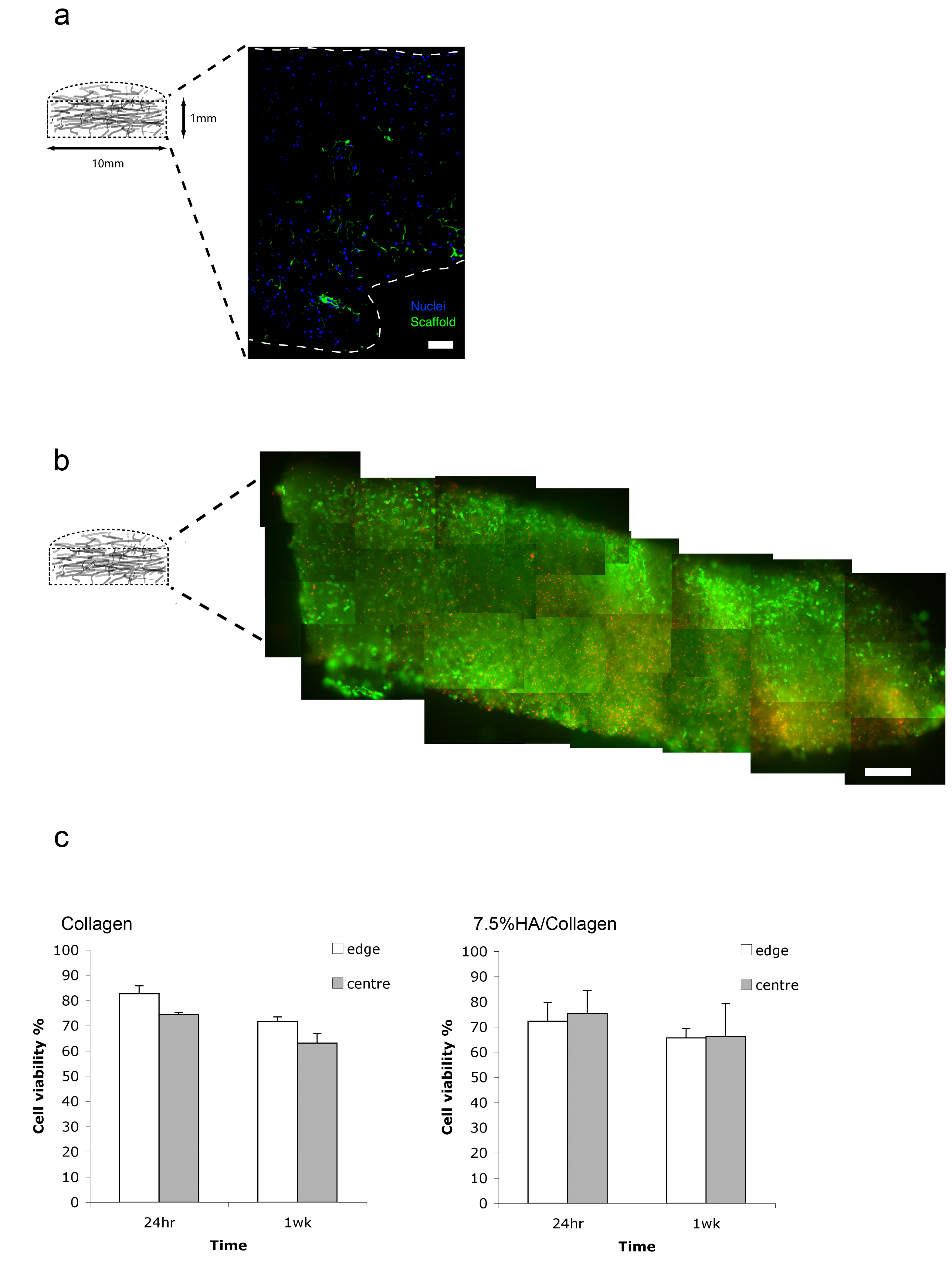

Supplement: Figure S3 — Seeding and viability of cells in 3D scaffolds. (a) Full depth seeding of 3T3-L1 preadipocyte cell suspensions in collagen scaffold (bar = 100 µm). (b) Viability assessment within cross section of 3T3-L1 seeded collagen scaffold following 12 days of culture (green = calcein AM, red = PI). (c) Mean 3T3-L1 viability at indicated times within collagen and 7.5%HA/col scaffolds. Live and dead cells were counted within edge (to a depth <185 µm) and centre regions using ImageJ software. Error bars represent standard deviation. (TIF) [file pone.0025661.s003.tif]

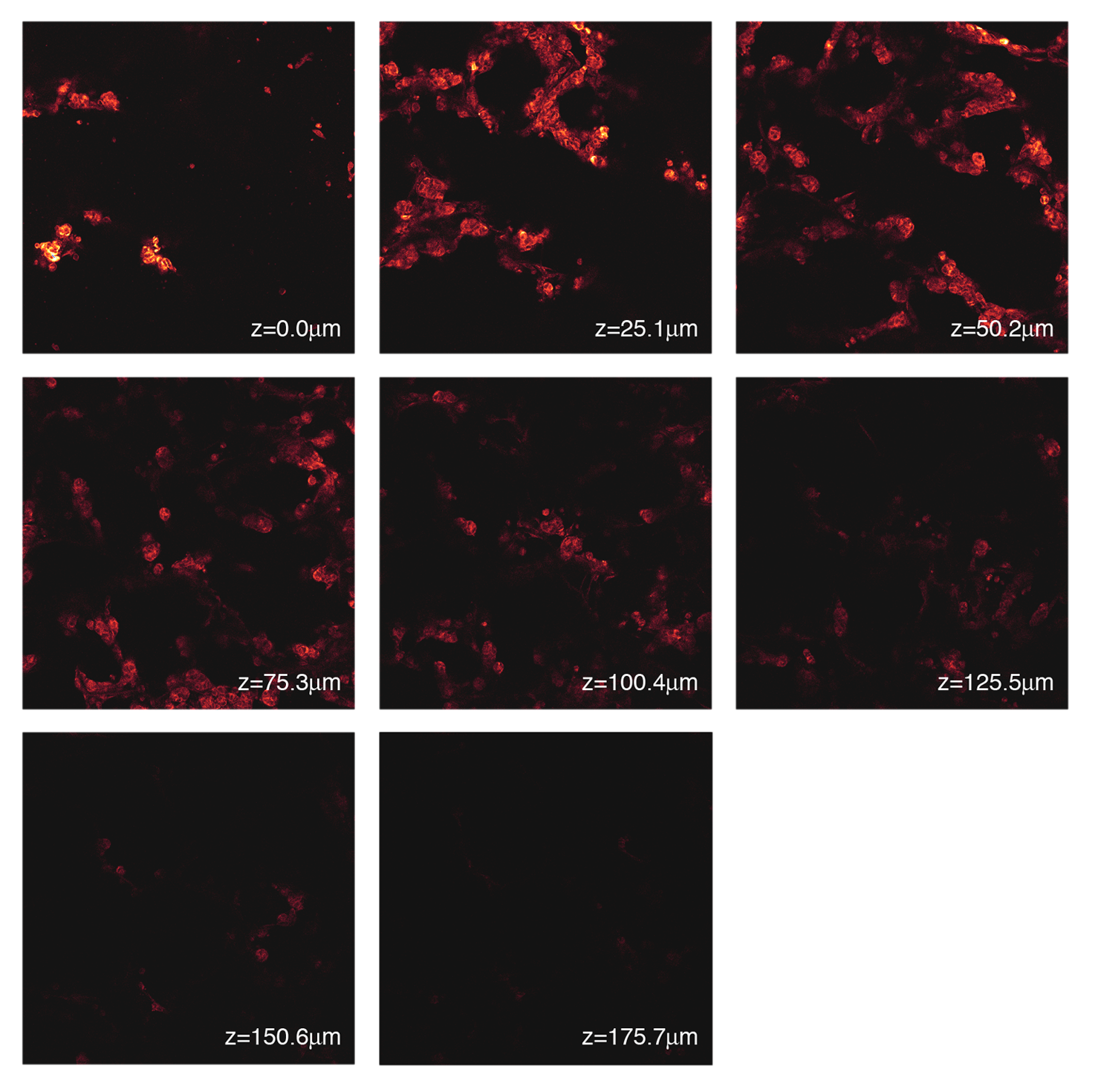

Supplement: Figure S4 — 3D epithelial organoid formation towards the scaffold surface. Confocal live-cell images at indicated depths (z dimension) of KIM-2 mono-cultures in 7.5%HA/col scaffold following 1 wk of culture in maintenance media (cells labelled with cell tracker red with look-up table imaging, Leica confocal software) (TIF) [file pone.0025661.s004.tif]

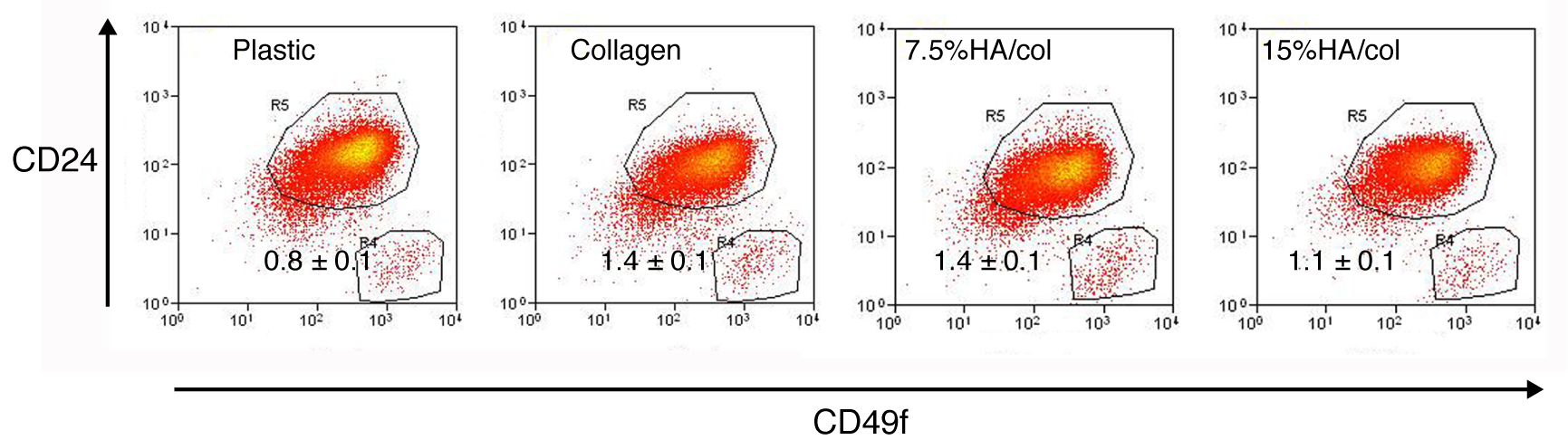

Supplement: Figure S5 — Analysis of luminal and basal mammary epithelial cells cultured on fabricated films. Representative FACS dot plots of KIM-2 cells cultured for one week on plastic or on 2D films of collagen, 7.5%HA/col or 15%HA/col and stained with CD24 and CD49f antibodies to separate luminal (CD24high CD49fmed) and basal (CD24med CD49fhigh) populations. The mean % +/- SD of basal cells within the total cell population in each condition is shown. (TIF) [file pone.0025661.s005.tif]

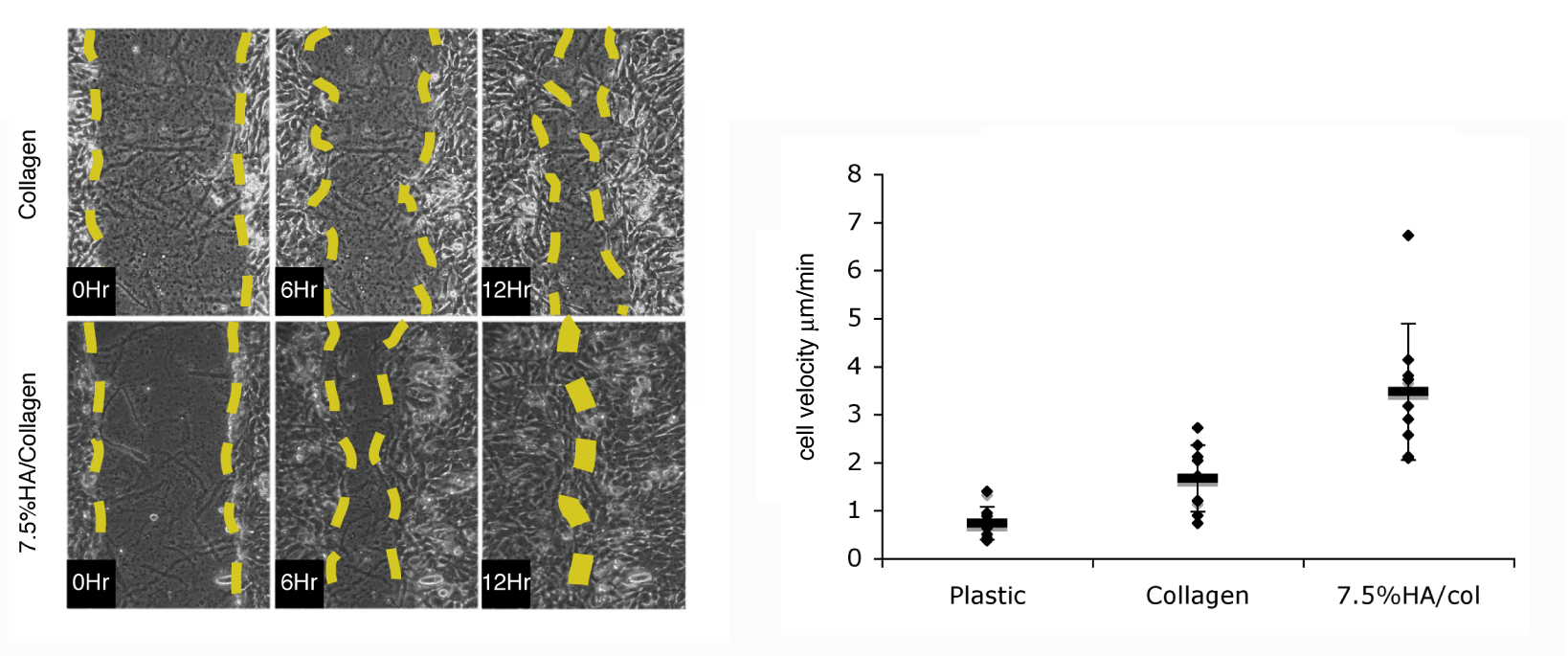

Supplement: Figure S6 — Cell migration analysis on fabricated films by wound closure assay. Representative images of scratch assays at indicated times on collagen and 7.5%HA/col films. Yellow line indicates migratory front of cells. Plotted velocities of KIM-2 cells µm/min−1. (TIF) [file pone.0025661.s006.tif]
